# Does paraspinal muscle morphometry predict functional status and re-operation after lumbar spinal surgery? A systematic review and meta-analysis

**DOI:** 10.1007/s00330-023-09548-6

**Published:** 2023-03-28

**Authors:** Gengyu Han, Haotian Wu, Jinyue Dai, Xinhang Li, Lihao Yue, Zheyu Fan, Qiaoyu Li, Qirui Shao, Yu Jiang, Weishi Li

**Affiliations:** 1 Department of Orthopaedics, Peking University Third Hospital, No. 49 NorthGarden Road, Haidian District, Beijing, 100191 China; 2 Engineering Research Center of Bone and Joint Precision Medicine, Ministry of Education, Beijing, China; 3 Beijing Key Laboratory of Spinal Disease Research, Beijing, China

**Keywords:** Treatment outcome, Paraspinal muscles, Surgical procedures, operative, Meta-analysis

## Abstract

**Objectives:**

Whether paraspinal muscle degeneration is related to poor clinical outcomes after lumbar surgery is still indistinct, which limits its clinical application. This study aimed to evaluate the predictive value of paraspinal muscle morphology on functional status and re-operation after lumbar spinal surgery.

**Methods:**

A review of the literature was conducted using a total of 6917 articles identified from a search of PubMed, EMBASE, and Web of Science databases through September 2022. A full-text review of 140 studies was conducted based on criteria including an objective assessment of preoperative paraspinal muscle morphology including multifidus (MF), erector spinae (ES), and psoas major (PS) in addition to measuring its relationship to clinical outcomes including Oswestry disability index (ODI), pain and revision surgery. Meta-analysis was performed when required metrics could be calculated in ≥ three studies, otherwise vote counting model was a good alternative to show the effect direction of evidence. The standardized mean difference (SMD) and 95% confidence interval (CI) were calculated.

**Results:**

A total of 10 studies were included in this review. Of them, five studies with required metrics were included in the meta-analysis. The meta-analysis suggested that higher preoperative fat infiltration (FI) of MF could predict higher postoperative ODI scores (SMD = 0.33, 95% CI 0.16–0.50, *p* = 0.0001). For postoperative pain, MF FI could also be an effective predictor for persistent low back pain after surgery (SMD = 0.17, 95% CI 0.02–0.31, *p* = 0.03). However, in the vote count model, limited evidence was presented for the prognostic effects of ES and PS on postoperative functional status and symptoms. In terms of revision surgery, there was conflicting evidence that FI of MF and ES could predict the incidence of revision surgery in the vote count model.

**Conclusion:**

The assessment of MF FI could be a viable method to stratify patients with lumbar surgery by the risk of severe functional disability and low back pain.

**Key Points:**

• *The fat infiltration of multifidus can predict postoperative functional status and low back pain after lumbar spinal surgery.*

• *The preoperative evaluation of paraspinal muscle morphology is conducive for surgeons.*

**Supplementary Information:**

The online version contains supplementary material available at 10.1007/s00330-023-09548-6.

## Introduction

Spine surgery is a common adjunct treatment for degenerative spinal diseases, which has increased significantly in recent years [1]. However, this is associated with a growth in the frequency of inferior postoperative outcomes [2, 3]. Studies have revealed that the degeneration of paraspinal muscles, which can be generally found among elderly patients, is implicated in multiple degenerative lumbar pathologies [4–6].

Currently, the value of paraspinal muscle morphometry on image examination serving as a prognostic factor for several surgical disciplines including metastatic disease, trauma, and fracture is being unearthed [7–9]. Multifidus (MF), erector spinae (ES), and psoas major (PS), serving as primary extensor and flexor muscles, are generally quantified by the cross-sectional areas (CSA) and fat infiltration (FI) on magnetic resonance image (MRI). Although abundant work has been carried out to identify potential factors for the prognosis of spine surgery [10, 11], few unequivocal predictive factors related to paraspinal muscle have come to light.

Preoperative assessment of paraspinal muscle morphology may be conducive to identifying patients who tend to have unsatisfactory clinical outcomes and thus making precautionary measures in advance. Two systematic reviews have concluded the degree of preoperative paraspinal muscle degeneration in relation to several complications after spinal surgery [12, 13]. However, both of them could not conduct a meta-analysis because of high heterogeneity. Besides, they did not focus on the patients’ functional status and re-operation after surgery. Moreover, there exist conflicting results on whether paraspinal muscle morphology was associated with clinical outcomes in patients with lumbar surgery [14, 15]. In consequence, our systematic review and meta-analysis primarily aim to elucidate the predictive value of preoperative paraspinal muscle morphology on functional status, symptoms, and re-operation in patients with surgery for degenerative lumbar diseases.

## Materials and methods

### Search strategy and eligibility criteria

The Preferred Reporting Items for Systematic Reviews and Meta-Analyses (PRISMA) statement was used to structure this systematic review and meta-analysis. To retrieve interrelated articles, we conducted a search in the following three databases: PubMed, EMBASE, and Web of Science databases through September 2022. All fields were searched for these terms: “paraspinal muscle,” “paravertebral muscle,” “multifidus,” “erector spinae,” or “psoas major”; and “surgery,” “operative,” “clinical outcome,” or “functional status”; and “lumbar” or “lumbosacral.” Two authors assessed all abstracts and titles to rate adherence to review criteria. Inclusion criteria consisted of the following: (1) articles including adults with degenerative lumbar diseases; (2) assessment of any lumbar paraspinal muscle characteristic on MRI or computed tomography preoperatively; (3) assessment of any clinical outcomes after lumbar surgery; (4) analyzed the relationship between preoperative imaging data and postoperative outcomes; (5) articles were published in English. Studies were excluded if they included subjects < 18 years of age; assessed lumbar muscle through nonconventional MRI (such as functional MRI, MRS, and chemical-shift MRI) and ultrasonography; included only postsurgical subjects. Studies included randomized controlled trials, cohort studies, case–control studies, and case series.

### Assessment of risk of bias

We used Quality in Prognosis Studies (QUIPS), a widely accepted tool for evaluating the risk of bias in prognostic studies [16]. All articles meeting review criteria were evaluated independently for risk of bias by two authors, with any differences in assessment resolved by discussions until consensus was reached. The QUIPS contained 6 separate domains: study participation, study attrition, prognostic factor measurement, outcome measurement, study confounding, and statistical analysis and reporting. Each domain was judged as low, moderate, or high risk of bias, according to published criteria [17]. The overall risk of bias for an included study was defined as low risk with ≥ 4 low- and no high-risk domains, moderate risk with < 4 low- and no high-risk domains, and high risk with ≥ 1 high-risk domain [18].

### Data extraction

Two authors independently extracted the following information from included studies: study design, participant characteristics, details of assessments of preoperative lumbar muscle characteristics, and study results that were relevant to our research question. Any disagreement would be adjudicated by a third author.

### Measures and outcomes

The FI and atrophy of paraspinal muscles were evaluated by MRI or computed tomography. The parameters of high FI covered the increased percentage of the fat area or signal intensity of muscles, and the parameters of muscle atrophy included the declined area of total or lean paraspinal muscles. We defined the paraspinal extensor muscle (PEM) group as the integrity of MF and ES [19]. Oswestry disability index (ODI) scores were adopted to evaluate the postoperative functional disability [20]. Visual analogue scale (VAS) and numerical rating scale (NRS) were used to determine the postoperative pain at low back or legs [21]. The rate of revision surgery was also recorded.

For the applicability of synthesis, we have conducted subgroup analyses with a consistently measured paraspinal muscle and its method of morphology measure to reduce the possible heterogeneity among studies. Similar subgroup analyses were conducted in previous studies [19, 22].

### Data analysis and levels of evidence

The literature presented data in various forms, including the means with/without standard deviations (SDs) for continuous outcomes, odds ratios (ORs) for dichotomous outcomes, and correlation coefficient (r) in correlation analysis. In view of that, a standardized mean difference (SMD) was calculated from the reported means and SDs [23]. The SDs could also be estimated from *t* test value or *p* value with the degree of freedom, if not directly reported [24]. Other different metrics (ORs and correlation coefficients) were transformed into SMDs, for the feasibility of overall comparison among various metrics [25]. The effect size was defined as small, medium, large, or very large with a SMD at 0.10–0.34, 0.35–0.64, 0.65–1.19, or > 1.20 respectively [26].

Meta-analyses were performed to show the weighted effect size for homogenous comparisons when required metrics could be calculated in ≥ 3 studies according to previous studies [27, 28]. The analysis was completed by Revman 5.4.1 and Rstudio software [29]. The forest plots were used to report SMDs and 95% CIs for separate studies and weighted effects. The heterogeneity was evaluated by Cochran’s Q and *I*^2^ statistics tests. The fixed-effects model was selected when the heterogeneity was acceptable with *p* > 0.10 and *I*^*2*^ < 50%; otherwise, a random-effects model was fitted [27]. Publication bias was displayed by funnel plots and Egger’s test [23, 27].

When the meta-analysis was inappropriate due to the lack of metrics reported in < 3 studies, a vote-counting model was conducted according to previous studies, with the direction of effect [30–32]. The model followed the Cochrane and Synthesis without meta-analysis (SWiM) reporting guidelines [33, 34]. We performed a qualitative summary of evidence for lumbar muscle characteristics as predictors of postoperative outcomes. The model followed the definition for levels of evidence in previous reviews [35–37]: “strong” evidence was defined as consistent findings (≥ 75%), among which ≥ 2 studies were at low risk; “moderate” evidence was defined as consistent findings (≥ 75%), among which one study was at low-risk and ≥ 1 studies were at moderate- or high-risk; “limited” evidence was defined as consistent findings (≥ 75%), among which one study was at low- or moderate-risk or ≥ 2 studies were at high-risk; “conflicting” evidence was defined as inconsistent findings irrespective of study quality. One high-risk study was not considered in the vote-counting model. The effect direction plot displayed the direction of effect in the model [28, 34].

## Results

**Fig. 1:**
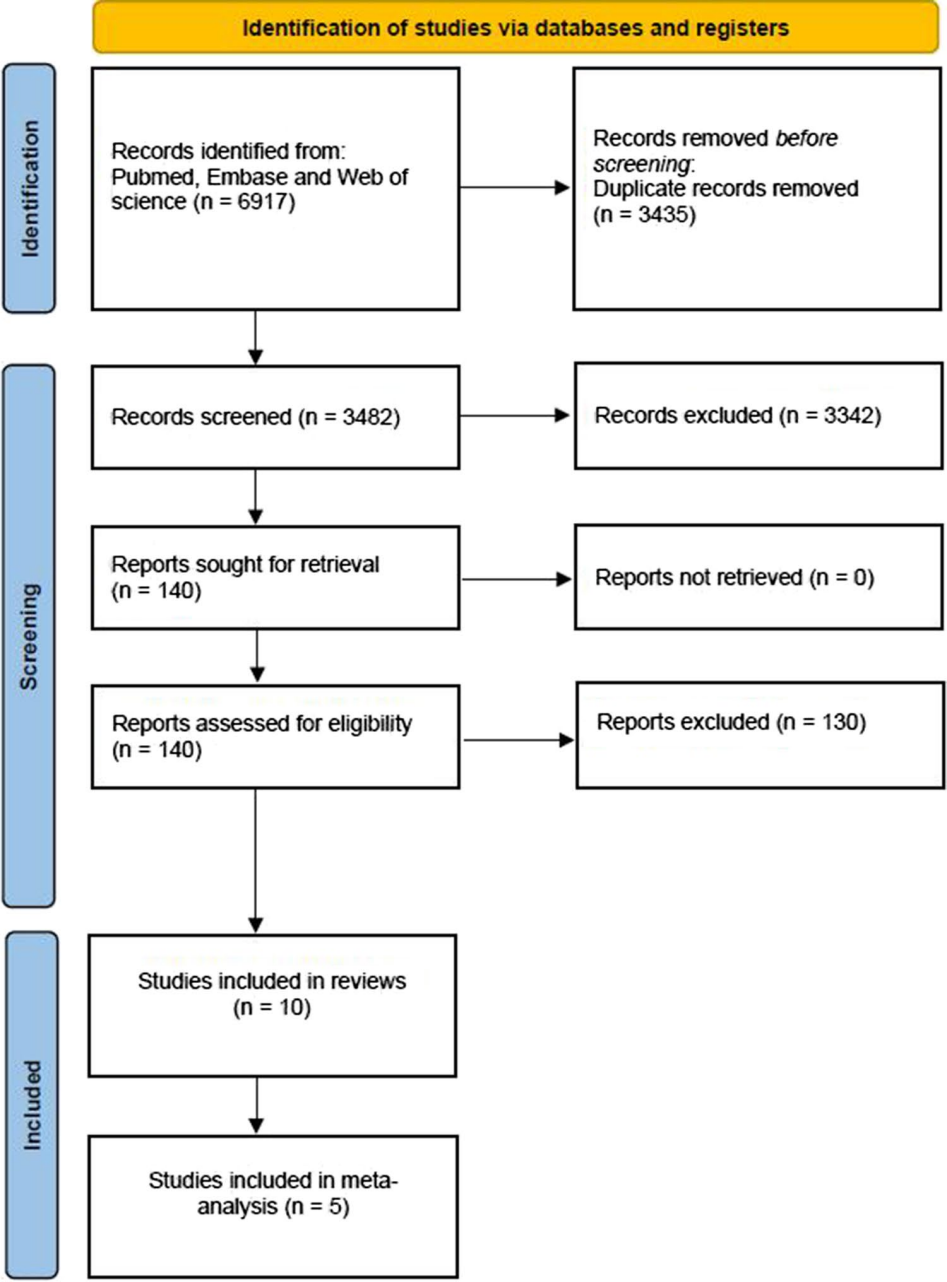
Flowchart of the study selection and inclusion process. A total of 6917 articles were identified from databases through September 2022. After the removal of duplicate records, 3482 studies were screened. Of these articles, 140 were eligible for full-text review. A total of 10 articles were included in the review and 5 articles were included in the meta-analysis

For the review of preoperative paraspinal muscle characteristics and their predictive value in clinical outcomes after spinal surgery, 6917 studies were identified through database searching. After the removal of duplicate records, 3482 studies were screened in our study. Of these articles, 140 were deemed to be eligible for full-text review. Finally, a total of ten articles were included. Of them, five studies with required metrics were included in the meta-analysis. The search flow diagram is shown in Fig. 1. The meta-analyses for the relationships between preoperative MF FI, postoperative ODI, and postoperative back pain were performed in a fixed-effects model since the heterogeneities were both acceptable (*p* = 0.13, *I*^*2*^ = 47% for ODI; *p* = 0.43, *I*^2^ = 0% for back pain).

### Study characteristics and risk of bias

**Fig. 2:**
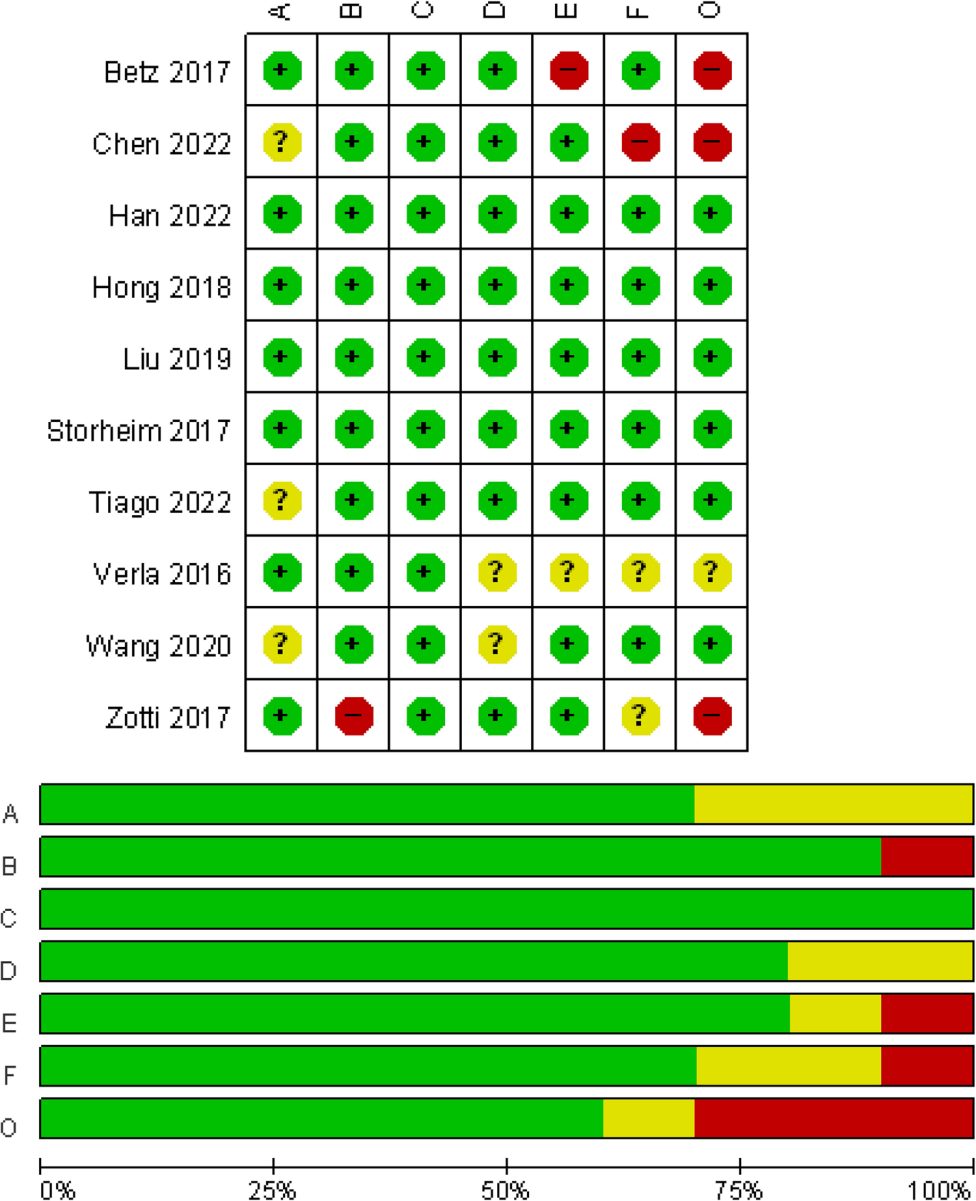
Risk of bias for the included studies. A = study participation, B = study attrition, C = prognostic factor measurement, D = outcome measurement, E = study confounding, F = statistical analysis and reporting, O = overall risk of bias. The overall risk of bias for an included study was defined as low risk with ≥ 4 low- and no high-risk domains, moderate risk with < 4 low- and no high-risk domains, and high risk with ≥ 1 high-risk domains

Of ten included studies, six studies investigated participants with lumbar disc herniation (LDH, *n* = 1) [38] or lumbar spinal stenosis (LSS, *n* = 5) [14, 15, 21, 39, 40], one reported spondylolisthesis [41], and three reported multiple lumbar degenerative diseases [42–44]. All articles examined the relationship between preoperative paraspinal muscle and postoperative symptoms and functional status and two articles examined the re-operation. All studies assessed the muscle morphology by MRI. There were five cohort studies, four case series, and one secondary analysis in the randomized controlled trial. After the assessments by QUIPS, there were six, one, and three studies considered to have a low, moderate, and high risk of bias, respectively (Fig. 2 and Supplementary table 1). The characteristics of the included studies are summarized in Table 1. In addition, publication bias was not observed in meta-analyses based on funnel plots and Egger’s test (Supplementary Figs. 1 and 2).

**Table 1:** Study characteristic of the included studies

Authors	Year	Design	Participants, n (male), age	Disease	Intervention	Radiology assessment	Outcome assessment	Metrics	Follow-up
Verla	2016	Retrospective case-series	257 (45.3%), 58.15 ± 14.96 y	Multiple degenerative lumbar diseases	LIF	Thickness of PS, by axial T1-MRI at level L1-5	ODI, VAS	Difference without SD and *p* value in subgroups	1.5 m
Storheim	2017	Secondary analysis in RCT	83 (47.6%), 41.0 ± 7.2 y	Chronic LBP and degenerative disc	total disc replacement	MF FI by axial T2- MRI (T1 if T2 was lacking) at level L3-4, L4-5 and L5-S1 FI grading 0 = < 20% of total cross-section (both sides) contains fat, 1 = 20–50% of cross-section contains fat, 2 = ≥ 50% of cross-section contains fat	ODI, VAS	Mean, SD, and P value	2 y
Zotti	2017	Prospective cohort	66 (37.9%), 67 (29–86) y	LSS	Spinal decompression	tCSA and FI of MF by axial and sagittal T1 and T2-MRI at level L4-5 tCSA: low = < 8.5 cm^2^, high = ≥ 8.5 cm^2^ Kader FI grade 0 = no fat streaks, 1 = < 10% of fat streaks, 2 = < 50% of fat streaks, 3 = > 50% of fat streaks	ODI	Patient number in subgroups and *p* value	2 y
Betz	2017	Prospective cohort	165 (44.8%), 73.8 ± 8.5 y	LSS	Decompression	PEM FI by axial T2-MRI (1.5 Tesla and 3 Tesla) at level L3 Goutallier/Fuchs FI grade 0 = no fatty streaks, 1 = some fatty streaks, 2 = important fatty streaks, but more muscle than fat, 3 = as much fat as muscle, 4 = more fat than muscle good paravertebral muscle quality: grade ≤ 1 bad paravertebral muscle quality: grade > 1	NRS, revision surgery	Patient number in subgroups, mean, SD, and *p* value	1 y
Hong	2018	Retrospective case-series	664 (63.4%), 41.2 ± 12.3 y	Single-level LDH	Microendoscopic discectomy surgery	MF FI by axial T2-MRI at level L3-4, L4-5 and L5-S1 Three-scale FI grading system: normal/ mild = FI < 10%, moderate = 10–50%, severe = > 50%, the most severe atrophy = left–right asymmetry in affected level	ODI, VAS, revision surgery	OR and *p* value	63.8 ± 20.0 (24–96) m
Liu	2019	Retrospective cohort	118 (52.5%), 59.8 ± 8.6 y	L4-5 single-segment DLSS	Decompression and fusion	MF FI by axial T2-MRI (3 Tesla) at the superior endplate level of S1 insignificant FI = < 25% of the tCSA significant FI = ≥ 25% of the tCSA	ODI, VAS	Mean, SD, and *p* value	18 m
Wang	2020	Retrospective case-series	69 (37.7%), 60.6 ± 9.4 y	Symptomatic LSS	PLIF	FI of MF and ES by axial T2-MRI (3 Tesla) at the inferior vertebral endplate of L4 FI (the ratio of tCSA minus fat-free CSA to tCSA)	ODI	Correlation coefficient and *p* value	6 m
Tiago	2022	Retrospective case-series	75 (32.0%), 52.3 y	Degenerative lumbar diseases	TLIF or PLIF	MF FI by axial T2-MRI at the interbody fusion vertebral level and adjacent levels Tamai FI grade: 0 = no intramuscular fat, 1 = some fatty streaks present, 2 = fat evident, but less than muscle tissue, 3 = amounts of fat equal to amount of muscle, 4 = more fat than muscle tissue	ODI, VAS, self-reported leg pain	Number of patients in subgroups mean without SD, and *p* value	2 y
Han	2022	Retrospective cohort	320 (65.0%), group 1: 60.7 ± 7.0 y; group 2: 59.9 ± 6.7 y	LSS	PLIF	MF FI by axial T2-MRI (3 Tesla) at L4 group 1 = FI ≥ 25%, group 2 = FI < 25%	ODI, VAS	Mean, SD, and *p* value	1 y
Chen	2022	Retrospective cohort	58 (48.3%), 51.4 ± 7.3 y	L4-5 single-segment spondylolisthesis	LLIF	MF CAS and FI by axial T1 and T2-MRI (1.5 Tesla) at level L4/5 muscle/vertebra CSA ratio grade: 1 = > 0.80, 2 = 0.60–0.80 and 3 = < 0.60 Kalichman FI grade: 1 = < 10% of tCSA, 2 = 11–50% of tCSA, 3 = > 50% of tCSA	ODI, VAS	Number of subgroup, mean, SD, and *p* value	20.6 ± 4.5 m

*RCT*, randomized controlled trial; *LBP*, low back pain; *LSS*, lumbar spinal stenosis; *LDH*, lumbar disc herniation; *LIF*, lumbar interbody fusion; *TLIF*, transforaminal lumbar interbody fusion; PLIF, posterior lumbar interbody fusion; *LLIF*, lateral lumbar interbody fusion; *MRI*, magnetic Resonance Imaging; *CT*, computed tomography; *MF*, multifidus; *ES*, erector spinae; *PS*, psoas major; *PEM*, paraspinal extensor muscle; *CSA*, cross-section area; *tCSA*, total cross-section area; *FI*, fatty infiltration; *ODI*, Oswestry disability index; *VAS*, visual analogue scale; *NRS*, numeric rating scale; *OR*, odds ratio; *CI*, confidence interval; *SD*, standard deviation

### Postoperative functional status and symptoms

#### Postoperative ODI

**Fig. 3:**
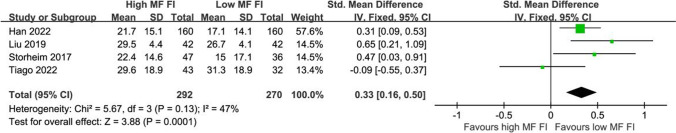
Forest plot of postoperative ODI between patients with high and low MF FI for lumbar surgery. Patients with high-grade preoperative MF FI had higher postoperative ODI scores, compared to those with low-grade MF FI (SMD = 0.33, 95% CI 0.16–0.50, *p* = 0.0001)

Eight studies investigated the relationship between preoperative paraspinal muscle morphology and postoperative ODI [14, 15, 38–43]. A meta-analysis including four studies with sufficient data in the fixed-effects model was performed for comparison in postoperative ODI scores between groups with high or low FI in MF [39, 40, 42, 43]. The meta-analysis in the fixed-effects model revealed that patients with a high grade of preoperative MF FI had higher postoperative ODI scores, compared to those with a low grade of MF FI (SMD = 0.33, 95% CI 0.16–0.50, *p* = 0.0001; Fig. 3).

**Fig. 4:**
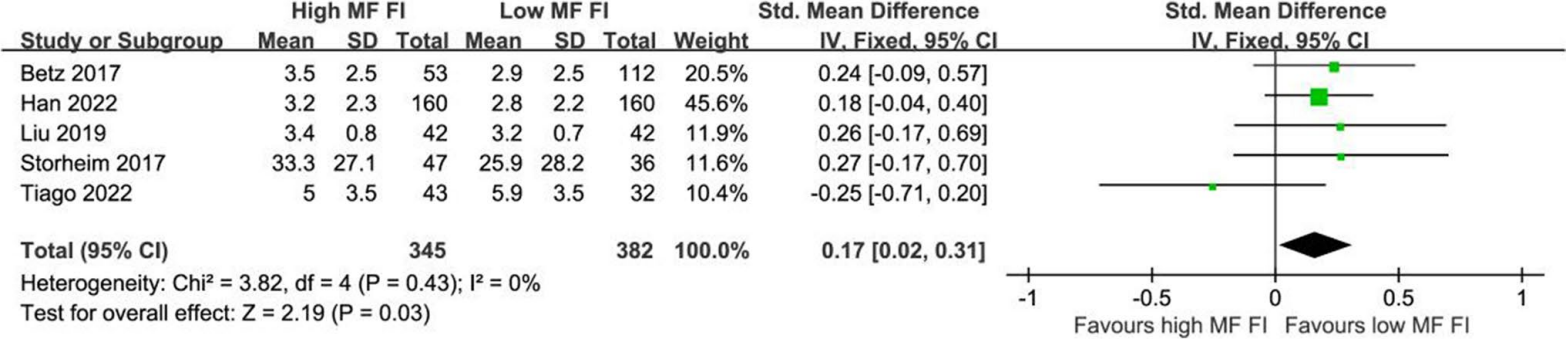
Forest plot of postoperative back pain between patients with high and low MF FI for lumbar surgery. Patients with higher preoperative FI of MF had greater postoperative LBP compared to those with low FI of MF (SMD = 0.17, 95%CI 0.02–0.31, *p* = 0.03)

In the vote-counting model, whether MF atrophy was related to postoperative ODI remained indistinct (Table 2). Zotti et al [14] found that greater tCSA (< 8.5 cm^2^) of MF at L4-5 predicted a larger improvement in ODI (SMD = 0.85, *p* = 0.006). Chen et al [41] reported that a higher muscle/vertebra CSA ratio (> 0.60) of MF at L4-5 was associated with more improvement in ODI (SMD = 0.55, *p* = 0.010). On the contrary, Wang et al [15] found that the lower scores and better improvement in postoperative ODI were not correlated to a higher tCSA of MF at L4 in patients with LSS after PLIF. As for ES, only one single study by Wang et al assessed the prognostic effect of CSA and FI on ODI [15]. Limited evidence revealed that FI of ES could predict the postoperative ODI after lumbar surgery, while atrophy of ES could not (Table 2 and Fig. 4). Moreover, PS thickness could be a possible indicator for change in ODI with limited evidence (Table 2). One study by Verla et al [44] measured the thickness of PS at each segment and revealed that more improvement (> 50%) in ODI was associated with greater psoas muscle thickness at L3-5 (*p* = 0.017) in patients with multiple degenerative lumbar diseases after PLIF.

**Table 2:** Levels of evidence for paraspinal muscle characteristics as predictors of postoperative ODI after lumbar surgery in vote counting model

Study	Support	Outcomes with metrics	Risk of bias	Level of evidence
Atrophy of MF can predict the postoperative ODI				Conflicting
Zotti 2017	Support	Patients with greater (> 8.5 cm^2^) MF CSA were more likely to obtain greater improvement (> 40%) in ODI (> 8.5 cm^2^: 13/19 vs. < 8.5 cm^2^: 15/47, *p* = 0.006) Standardized metrics: OR = 4.622, SMD = 0.85	High	
Chen 2022	Support	Greater MF CSA predicted higher ODI scores (*p* < 0.001) and more improvement (> 40%: *n* = 40, FI mean = 0.57, SD = 0.18 vs. < 40%: *n* = 18, FI mean = 0.48, SD = 0.11, *p* = 0.045) in patients with spondylolisthesis after LLIF Standardized metrics: improvement of ODI: SMD = 0.55 (95%CI − 0.02 to 1.11)	High	
Wang 2020	Not support	Higher MF tCSA failed to predict the lower level (*r* = − 0.005, *p* > 0.05) or better improvement (*r* = *− *0.218, *p* > 0.05) of ODI Standardized metrics: level: SMD = − 0.01; improvement: SMD = − 0.45	Low	
Atrophy of ES cannot predict the postoperative ODI				Limited
Wang 2020	Support	ES tCSA failed to predict the level (*r* = − 0.010, *p* > 0.05) or improvement (*r* = *− *0.135, *p* > 0.05) of ODI Standardized metrics: level: SMD = − 0.02, improvement: SMD = − 0.28	Low	
FI of ES can predict the postoperative ODI				Limited
Wang 2020	Support	Lower ES FI was correlated with a high level (*r* = 0.438, *p* < 0.01) although not correlated with better improvement (*r* = − 0.142, *p* > 0.05) of ODI Standardized metrics: level: SMD = 0.97, improvement: SMD = − 0.29	Low	
Atrophy of PS can predict thepostoperative ODI				Limited
Verla 2016	Support	Patients with greater improvement (> 50%) in ODI had greater PS thickness at level L3-4 (difference = 3.541 mm, *p* = 0.017) Standardized metrics: SMD cannot be calculated with limited information	Moderate	

*MF*, multifidus; *ES*, erector spinae; *PS*, psoas major; *CSA*, cross-section area; *tCSA*, total cross-section area; *FI*, fatty infiltration; *ODI*, Oswestry disability index; *OR*, odds ratio; *SMD*, standardized mean difference (SMD < 0 represented that the metric in patients with greater FI or CSA was lower than those with lower FI or CSA); *SD*, standard deviation; *95%CI*, 95% confidence interval; *r*, correlation coefficient; *p*, *p* value

#### Postoperative pain

Eight studies evaluated the association between postoperative pain and FI of paraspinal muscles [21, 38–44]. A meta-analysis including five studies with sufficient data in the fixed-effects model was conducted for comparison in low back pain (LBP) between groups with high or low FI in MF [21, 39, 40, 42, 43]. The meta-analysis in the fixed-effects model reported that the patients with higher preoperative FI of MF had greater postoperative LBP compared to those with low FI of MF (SMD = 0.17, 95%CI 0.02–0.31, *p* = 0.03; Fig. 4).

In the vote counting model, whether FI of MF could predict persistent leg pain remained conflicting (Table 3). Although some studies by Hong et al [38], Liu et al [40], and Chen et al [41] showed that MF FI failed to predict the improvement in VAS for leg pain, Tiago et al [42] uncovered the predictive evidence of lower MF FI for greater relief of self-reported leg pain post surgeries (grades 3–4: 35/43 vs. grades 1–2: 31/32, SMD =  − 1.09, *p* = 0.04). Han et al [39] also discovered a higher VAS for leg pain in LSS patients with higher MF FI (≥ 25% tCSA: n = 160, mean = 2.8, SD = 2.4) than lower MF FI (< 25% tCSA: *n* = 160, mean = 2.3, SD = 2.5) after PLIF (SMD = 0.20, *p* = 0.039). Besides, limited evidence showed that the atrophy of PS could predict persistent LBP (Table 3)**.** Verla et al [44] found that more improvement (> 50%) in VAS was associated with a greater PS thickness at L2-3 (*p* = 0.032), L3-4 (*p* = 0.043), and L4-5 (*p* = 0.022) in patients with multiple degenerative lumbar diseases after PLIF.

**Table 3:** Levels of evidence for paraspinal muscle characteristics as predictors of postoperative pain after lumbar surgery in vote counting model

Study	Support	Outcomes with metrics	Risk of bias	Level of evidence
FI of MF can predict the persistent leg pain				Conflicting
Tiago 2022	Support	Regarding the ‘worst’ level (level with the highest grade of MF FI), higher MF FI was associated with a lower rate of self-reported relief in leg pain (grades 3–4: 35/43 vs. grades 1–2: 31/32, *p* = 0.04) Standardized metrics: OR** = **0.14, SMD = − 1.09	Low	
Han 2022	Support	Patients with smaller (< 25%) MF FI had lower VAS (*n* = 160, mean = 2.3, SD = 2.5) for leg than those with MF FI ≥ 25% (*n* = 160, mean = 2.8, SD = 2.4) (*p* = 0.039) Standardized metrics: SMD = 0.20 (95%CI − 0.02 to 0.42)	Low	
Liu 2019	Not support	Patients with insignificant (< 25%: *n* = 41, mean = 3.1, SD = 0.6) and significant (≥ 25%: *n* = 41, mean = 3.2, SD = 0.5) MF FI had similar VAS-leg at 6 months after a propensity score matching including 82 patients (*p* = 0.423) Patients with insignificant (< 25%: *n* = 42, mean = 3.2, SD = 0.7) and significant (≥ 25%: *n* = 42, mean = 3.2, SD = 0.6) MF FI had similar VAS-leg at 18 months after a propensity score matching including 84 patients (*p* = 0.950) Standardized metrics: at 6 months: SMD = 0.18 (95%CI − 0.25 to 0.61), at 18 months: SMD = 0 (95%CI − 0.43 to 0.43)	Low	
Chen 2022	Not support	MF FI failed to predict VAS-leg (*p* = 0.213) and improvement (*p* > 0.05) in patients with spondylolisthesis after LLIF Standardized metrics: SMD cannot be calculated with limited information	High	
Hong 2018	Not support	MF FI failed to predict the rate of > 30% improvement of VAS-leg (*p* > 0.05) Standardized metrics: SMD cannot be calculated with limited information	Low	
Atrophy of PS can predict the persistent LBP				Limited
Verla 2016	Support	Patients with greater improvement (> 50%) in VAS had greater PS thickness (difference at level L2-3 = 4.015 mm, *p* = 0.032; at L3-4 = 4.128 mm, *p* = 0.043; at L4-5 = 5.012, *p* = 0.022) Standardized metrics: SMD cannot be calculated with limited information	Moderate	

*MF*, multifidus; *PS*, psoas major; *FI*, fatty infiltration; *VAS*, visual analogue scale; *LBP*, low back pain; *OR*, odds ratio; *SMD*, standardized mean difference (SMD < 0 represented that the metric in patients with greater FI or CSA was lower than those with lower FI or CSA); *SD*, standard deviation; *95%CI*, 95% confidence interval; *p*, *p* value

#### Re-operation

In the vote counting model, conflicting evidence existed in the prognostic value of MF and ES on the re-operation rate (Table 4). Betz et al [21] quantified the preoperative quality of the PEM group by the degree of fatty degeneration (according to the Goutallier classification) at L3. They found that there was no difference in revision surgery between symptomatic LSS patients who underwent revision surgery with good or bad muscle quality (SMD =  − 0.17, *p* = 0.55). Whereas Hong et al [38] confirmed that MF atrophy (OR = 2.095, SMD = 0.41, *p* = 0.01) at the involved level according to a three-scale grading system on MRI was a statistically significant predictor of a need for re-operation in patients with microendoscopic discectomy for single-level LDH.

**Table 4:** Levels of evidence for paraspinal muscle characteristics as predictors of re-operation after lumbar surgery in vote counting model

Study	Support	Outcomes with metrics	Risk of bias	Level of evidence
FI of PEM can predict the incident of revision surgery				Conflicting
Hong 2018	Support	Patients with higher MF FI had higher rates of revision surgeries (OR = 2.095, *p* = 0.001) Metrics: SMD = 0.41	Low	
Betz 2017	Not support	Patients with lower (grades 0–1: 11/112) or higher (grades 2–4: 4/53) PEM FI had a similar rate of revision surgery (*p* = 0.55) Standardized metrics: OR = 0.74, SMD = − 0.17	High	

*PEM*, paraspinal extensor muscle; *FI*, fatty infiltration; *OR*, odds ratio; *SMD*, standardized mean difference (SMD < 0 represented that the metric in patients with greater FI or CSA was lower than those with lower FI or CSA); *p*, *p* value

**Fig. 5:**
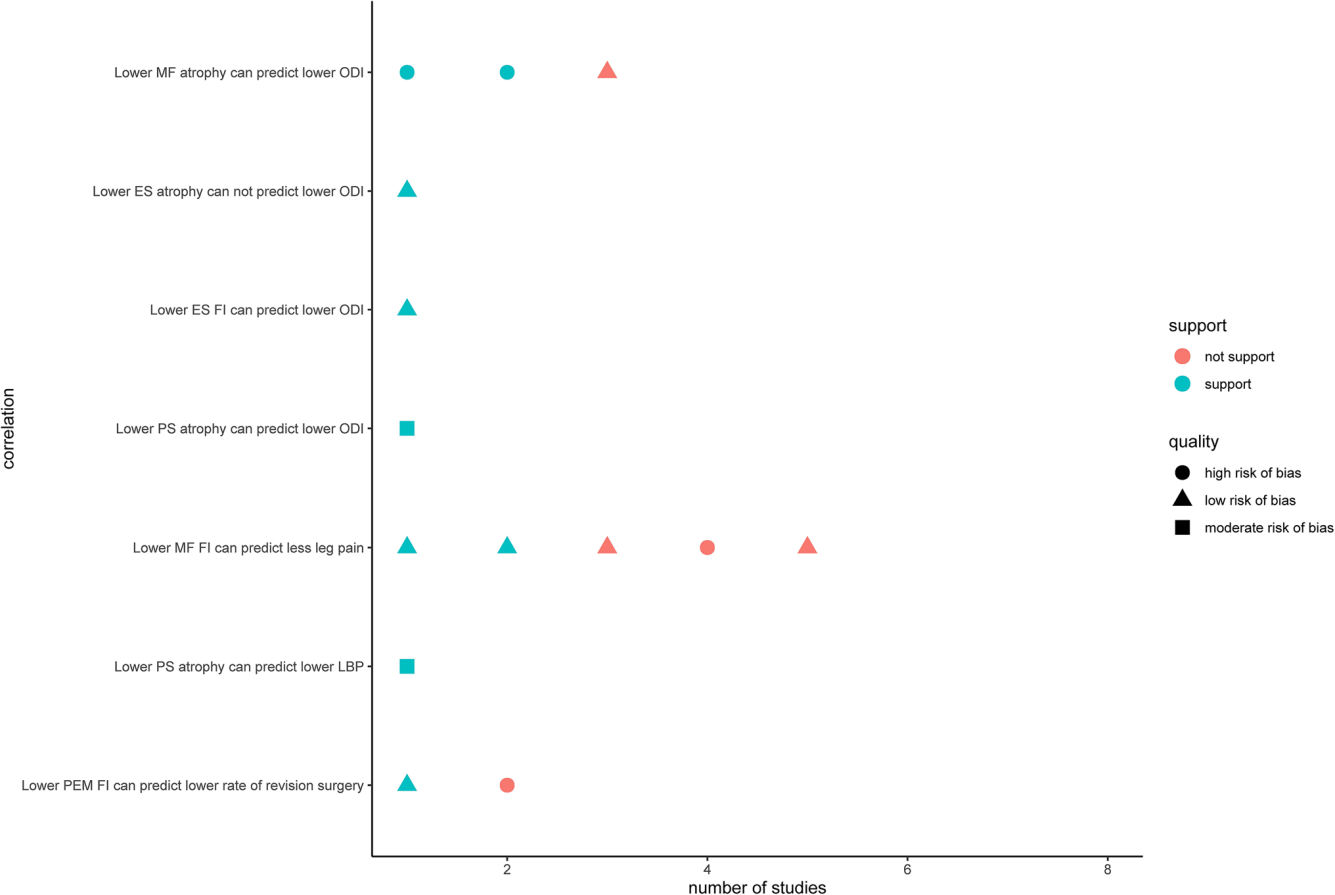
Effect direction plot for vote counting model. Evidence remained conflicting in terms of whether MF atrophy could predict ODI, whether MF FI could predict leg pain, and whether PEM FI could predict revision surgery. Besides, evidence remained limited in terms of whether ES atrophy, ES FI, or PS atrophy could predict ODI, and whether PS atrophy could predict low back pain

In our analysis, all of the pieces of evidence in the vote-counting model were shown in an effect direction plot (Fig. 5).

## Discussion

Paraspinal muscle degeneration can be visibly characterized by a muscle atrophy and an increased FI. Multiple studies have considered paraspinal muscle degeneration as a prognostic factor for surgical disciplines [7–9]. However, whether paraspinal muscle degeneration is related to a poor improvement of functional status after lumbar surgery is still indistinct. This is the first study that investigated the predictive value of three main back muscles on postoperative functional status and re-operation.

This systematic review and meta-analysis included ten studies providing evidence for relationships between lumbar muscle characteristics and postoperative functional status and symptoms. First, the meta-analysis found an association between MF FI and postoperative ODI. Previous studies have also demonstrated the relationship between MF FI and postoperative complications, which was accordant to our findings [12, 13]. The reason why patients with higher MF FI have poorer ODI might be interpreted by the fact that increased muscle FI is correlated to poorer muscle function and low physical activity [45–48]. Besides, only two studies assessed ES and PS. As a result, the evidence for ES and PS as prognostic factors is limited.

In terms of postoperative pain, higher MF FI could predict persistent LBP after lumbar surgery in our meta-analysis. Compared to other paraspinal muscles, MF was more closely related to the vertebral plate and spinous process [49]. Thus, the ability of MF to stabilize and control the movement of the lumbar spine may be a crucial factor in the development of symptoms, since those with severer symptoms have lower activity in MF [50]. In addition, our findings suggested that FI, not atrophy, was a good predictor for postoperative outcomes, which would be helpful for clinical decision-making. The reason might be that the relationship between atrophy and muscle function was not as significant as that of FI. A study demonstrated that FI of paraspinal muscles, rather than CSA, remained a significant predictor of extensor strength in multivariate regression [45]. Moreover, we found limited evidence that showed that the atrophy of PS could predict persistent LBP. As PS is an indicator of sarcopenia and correlates to clinical outcomes in several surgeries [7, 9], it may be a potential factor for predicting LBP.

In our study, two articles examined the relationship between preoperative paraspinal muscle and the re-operation. There was conflicting evidence that MF and ES could predict the re-operation rate in lumbar surgery. The implementation of revision surgery may be due to the failure to achieve osseous fusion, complications resulting from surgical implants, adjacent segment diseases, and persistent pain, part of which were associated with paraspinal muscle degeneration [13, 51]. Consequently, considering the small amount of included studies, the relationship between paraspinal muscles and re-operation needs more high-quality studies.

There are some limitations in this systematic review and meta-analysis. First, the amount of included studies was relatively small, which might produce bias. However, the included studies have been more than previous systematic reviews that investigated factors of clinical outcomes in degenerative lumbar spine diseases [19, 23]. Besides, although the funnel plots and Egger’s test indicated no evidence for publication bias in our study, the publication bias assessment based on a small number of included studies might be unreliable. Second, heterogeneity existed in our study, such as diseases and operations. Previous reviews investigating paraspinal muscle degeneration could not perform a meta-analysis due to high heterogeneity [12, 13]. However, in our review, we made subgroup analyses to reduce the heterogeneity (*I*^2^ < 50%) and made it possible for meta-analyses.

In conclusion, FI of MF could be a predictive factor of postoperative ODI and LBP. Whereas, for postoperative leg pain, MF FI could not be a predictor with conflicting evidence. Besides, limited evidence was presented for the prognostic effects of ES and PS on postoperative functional status and symptoms. Additionally, there was conflicting evidence that FI of MF and ES could predict the incidence of revision surgery. The results suggested that the assessment of paraspinal muscle degeneration could be a viable method to stratify patients by risk of postoperative functional status and pain to some extent. A rehabilitation approach focused on training MF before and after surgery might improve clinical outcomes. Considering that limited studies were included and conflicting or limited evidence also existed in this study, more research needs to focus on this field for assisting surgeons to predict the prognosis.

## Supplementary Information

Below is the link to the electronic supplementary material.

Supplementary file1 (PDF 368 kb)

## Abbreviations

CI: Confidence interval
CSA: Cross-sectional areas
DDD: Degenerative disc disease
ES: Erector spinae
FI: Fat infiltration
JBI: Joanna Briggs Institute
LBP: Low back pain
LDH: Lumbar disc herniation
LLIF: Lateral lumbar interbody fusion
LSS: Lumbar spinal stenosis
MF: Multifidus
NRS: Numerical rating scale
ODI: Oswestry disability index
OR: Odd ratio
PEM: Paraspinal extensor muscle
PLIF: Posterior lumbar interbody fusion
PRISMA: Preferred reporting items for systematic reviews and meta-analyses
PS: Psoas major
SMD: Standardized mean difference
SWiM: Synthesis without meta-analysis
tCSA: Total cross-sectional area
TLIF: Transforaminal lumbar interbody fusion
VAS: Visual analogue scale

## Declarations

### Guarantor

The scientific guarantor of this publication is Weishi Li.

### Statistics and biometry

No complex statistical methods were necessary for this paper.

### Methodology

• Not applicable

• Not applicable

• Performed at one institution
