# Supplementary material for: Does paraspinal muscle morphometry predict functional status and re-operation after lumbar spinal surgery? A systematic review and meta-analysis
Source: Eur Radiol. 2023 Mar 28;33(8):5269–81. doi: 10.1007/s00330-023-09548-6 (PMC10326143; doi:10.1007/s00330-023-09548-6)
Supplement: Supplementary file 1 — Supplementary file1 (PDF 368 kb) [file 330_2023_9548_MOESM1_ESM.pdf]

Supplementary Table 1. Risk of bias by QUIPS tools.

	Verla 2016	Storheim 2017	Zotti 2017	Betz 2017	Hong 2018	Liu 2019	Wang 2020	Tiago 2022	Han 2022	Chen 2022
**Study Participation**										
Source of target population	yes	yes	yes	yes	yes	yes	yes	yes	yes	yes
Method used to identify population	yes	yes	yes	yes	yes	yes	yes	yes	yes	yes
Recruitment period	yes	yes	yes	yes	yes	yes	yes	yes	yes	yes
Place of recruitment	yes	yes	yes	yes	yes	yes	yes	yes	yes	yes
Inclusion and exclusion criteria	yes	yes	yes	yes	yes	yes	yes	yes	yes	yes
Adequate study participation	yes	yes	yes	yes	yes	yes	no	no	yes	no
Baseline characteristics	yes	yes	yes	partial	yes	yes	yes	yes	yes	yes
**Summary Study participation (risk of bias)**	**low**	**low**	**low**	**low**	**low**	**low**	**moderate**	**moderate**	**low**	**moderate**
**Study Attrition**										
Proportion of baseline sample available for analysis	yes	yes	unsure	yes	yes	yes	yes	yes	yes	yes
Attempts to collect information on participants who dropped out	yes	yes	unsure	yes	yes	yes	yes	yes	yes	yes
Reasons and potential impact of subjects lost to follow-up	yes	yes	unsure	yes	yes	yes	yes	yes	yes	yes
Outcome and prognostic factor information on those lost to follow-up	unsure	unsure	unsure	unsure	unsure	unsure	unsure	unsure	unsure	unsure
**Study Attrition Summary (risk of bias)**	**low**	**low**	**high**	**low**	**low**	**low**	**low**	**low**	**low**	**low**
**Prognostic Factor Measurement**										
Definition of the PF	yes	yes	yes	yes	yes	yes	yes	yes	yes	yes
Valid and Reliable Measurement of PF	yes	yes	yes	yes	yes	yes	yes	yes	yes	yes
Proportion of data on PF available for analysis	yes	yes	yes	yes	yes	yes	yes	yes	yes	yes
Method used for missing data	yes	yes	yes	yes	yes	yes	yes	yes	yes	yes

**PF Measurement Summary (risk of bias)**	**low**	**low**	**low**	**low**	**low**	**low**	**low**	**low**	**low**	**low**
**Outcome Measurement**										
Definition of the Outcome	yes	yes	yes	yes	yes	yes	yes	yes	yes	yes
Valid and Reliable Measurement of Outcome	no	yes	yes	yes	yes	yes	no	yes	yes	yes
Method and Setting of Outcome Measurement	yes	yes	yes	yes	yes	yes	yes	yes	yes	yes
**Outcome Measurement Summary (risk of bias)**	**moderate**	**low**	**low**	**low**	**low**	**low**	**moderate**	**low**	**low**	**low**
**Study Confounding**										
Important Confounders Measured	yes	yes	yes	unsure	yes	yes	yes	yes	yes	yes
Definition of the confounding factor	unsure	yes	yes	unsure	yes	yes	unsure	unsure	yes	unsure
Valid and Reliable Measurement of Confounders	yes	yes	yes	unsure	yes	yes	yes	yes	yes	yes
Method and Setting of Confounding Measurement	yes	yes	yes	unsure	yes	yes	yes	yes	yes	yes
Method used for missing data	yes	yes	yes	yes	yes	yes	yes	yes	yes	yes
Appropriate Accounting for Confounding	unsure	yes	yes	unsure	yes	yes	yes	yes	yes	yes
**Study Confounding Summary (risk of bias)**	**moderate**	**low**	**low**	**high**	**low**	**low**	**low**	**low**	**low**	**low**
**Statistical Analysis and Reporting**										
Presentation of analytical strategy	unsure	yes	yes	yes	yes	yes	yes	yes	yes	no
Model development strategy	yes	yes	unsure	yes	yes	yes	yes	yes	yes	yes
Reporting of results	yes	yes	yes	yes	yes	yes	yes	yes	yes	yes
**Statistical Analysis and Presentation Summary**	**moderate**	**low**	**moderate**	**low**	**low**	**low**	**low**	**low**	**low**	**high**
**Overall bias in 6 domains**	**moderate**	**low**	**high**	**high**	**low**	**low**	**low**	**low**	**low**	**high**

Supplementary table 2. Data for studies in meta-analysis of postoperative ODI between patients with high and low MF FI for lumbar surgery.

Study	High FI of MF			Low FI of MF		
	Characteristics	Mean ODI (SD)	Sample size	Characteristics	Mean ODI (SD)	Sample size
Storheim 2017	FI > 20%	22.4 (14.6)	47	FI < 20%	15.0 (17.1)	36
Liu 2019	FI ≥ 25%	29.5 (4.4)	42	FI < 25%	26.7 (4.1)	42
Han 2022	FI > 25%	21.7 (15.1)	160	FI < 25%	17.1 (14.1)	160
Tiago 2022	Grade 0 = no intramuscular fat, I = some fatty streaks, II = fat less than muscle tissue	29.6 (18.9*)	43	Grade III = fat equal to amount of muscle, grade IV = more fat than muscle tissue	31.3 (18.9*)	32

* Estimated from means, precise P value and degree of freedom.

Supplementary table 3. Data for studies in meta-analysis of postoperative back pain between patients with high and low MF FI for lumbar surgery.

**Study**	**High FI of MF**			**Low FI of MF**		
	**Characteristics**	**Mean score (SD)**	**Sample size**	**Characteristics**	**Mean score (SD)**	**Sample size**
Betz 2017	Goutallier FI grade ≥ 2	NRS = 3.5 (2.5)	53	Goutallier FI grade < 2	NRS = 2.9 (2.5)	112
Storheim 2017	FI > 20%	VAS = 33.3 (27.1)	47	FI >20%	VAS = 25.9 (28.2)	36
Liu 2019	FI ≥ 25%	VAS = 3.4 (0.8)	42	FI < 25%	VAS = 3.2 (0.7)	42
Han 2022	FI > 25%	VAS = 3.2 (2.3)	160	FI < 25%	VAS = 2.8 (2.2)	160
Tiago 2022	Grade 0 = no intramuscular fat, I = some fatty streaks, II = fat less than muscle tissue	VAS = 5 (3.5*)	43	Grade III = fat equal to amount of muscle, grade IV = more fat than muscle tissue	VAS = 5.9 (3.5*)	32

*Estimated from means, precise P value and degree of freedom.

(1)

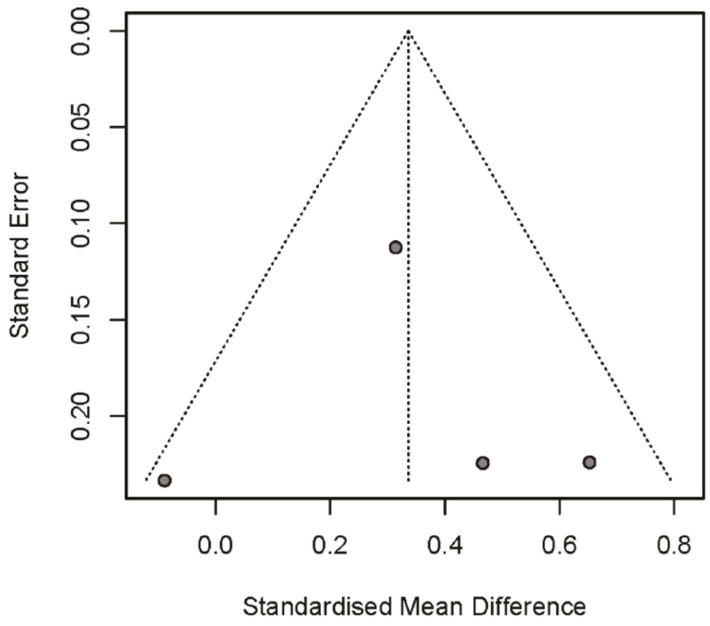

(2)

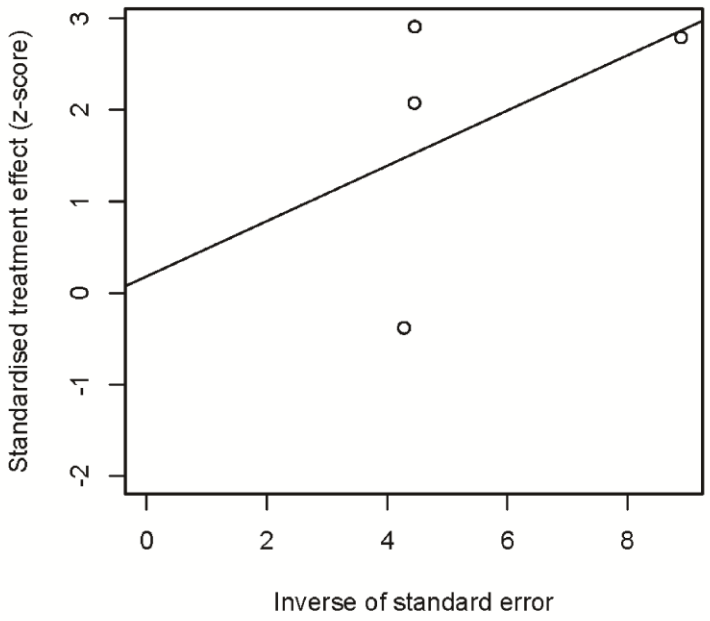

Supplementary figure 1. Funnel plot and Egger's test for meta-analysis of postoperative ODI between patients with high and low MF FI for lumbar surgery. The funnel plot and Egger's test were conducted by Rstudio. P value in Egger's test = 0.95.

(1) (2)

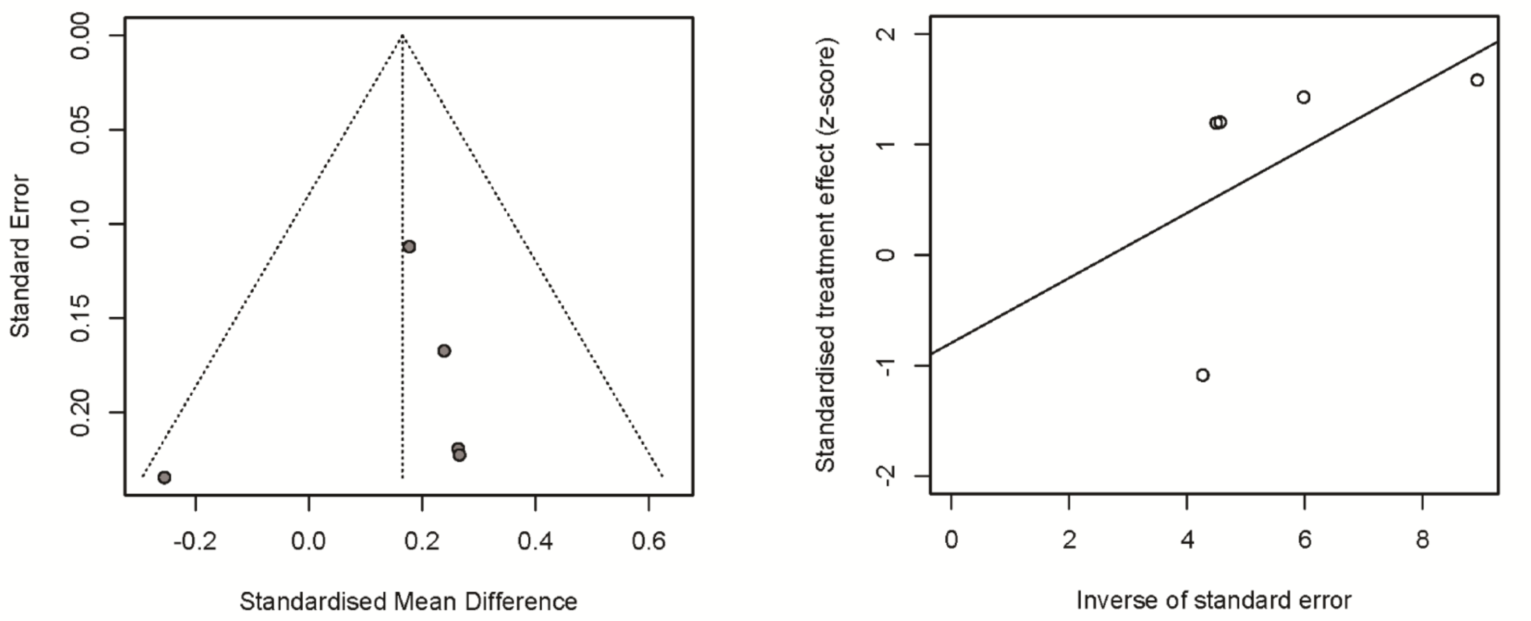

Supplementary figure 2. Funnel plot and Egger's test for meta-analysis of postoperative back pain between patients with high and low MF FI for lumbar surgery. The funnel plot and Egger's test were conducted by Rstudio. P value in Egger's test = 0.66.
